# Preserved Inhibitory Control Deficits of Overweight Participants in a Gamified Stop-Signal Task: Experimental Study of Validity

**DOI:** 10.2196/25063

**Published:** 2021-03-12

**Authors:** Philipp Alexander Schroeder, Johannes Lohmann, Manuel Ninaus

**Affiliations:** 1 Department of Psychology Clinical Psychology and Psychotherapy University of Tübingen Tübingen Germany; 2 Department of Computer Science Cognitive Modeling University of Tübingen Tübingen Germany; 3 Department of Psychology University of Innsbruck Innsbruck Austria; 4 Leibniz-Institut für Wissensmedien Tübingen Germany; 5 LEAD Graduate School and Research Network University of Tübingen Tübingen Germany

**Keywords:** gamification, inhibitory control, response inhibition, overweight, BMI, stop-signal task, mental health, games, overweight

## Abstract

**Background:**

Gamification in mental health could increase training adherence, motivation, and transfer effects, but the external validity of gamified tasks is unclear. This study documents that gamified task variants can show preserved associations between markers of behavioral deficits and health-related variables. We draw on the inhibitory control deficit in overweight populations to investigate effects of gamification on performance measures in a web-based experimental task.

**Objective:**

This study tested whether associations between inhibitory control and overweight were preserved in a gamified stop-signal task (SST).

**Methods:**

Two versions of an adaptive SST were developed and tested in an online experiment. Participants (n=111) were randomized to 1 of the 2 task variants and completed a series of questionnaires along with either the gamified SST or a conventional SST. To maximize its possible effects on participants’ inhibitory control, the gamified SST included multiple game elements in addition to the task itself and the stimuli. Both variants drew on the identical core mechanics, but the gamified variant included an additional narrative, graphical theme, scoring system with visual and emotional feedback, and the presence of a companion character. In both tasks, food and neutral low-poly stimuli were classified based on their color tone (go trials), but responses were withheld in 25% of the trials (stop trials). Mean go reaction times and stop-signal reaction times (SSRT) were analyzed as measures of performance and inhibitory control.

**Results:**

Participants in the gamified SST had longer reaction times (803 [SD 179] ms vs 607 [SD 90] ms) and worse inhibitory control (SSRT 383 [SD 109] ms vs 297 [SD 45] ms). The association of BMI with inhibitory control was relatively small (*r*=.155, 95% CI .013-.290). Overweight participants had longer reaction times (752 [SD 217] ms vs 672 [SD 137] ms) and SSRTs (363 [SD 116] ms vs 326 [SD 77] ms). Gamification did not interact with the effect of overweight on mean performance or inhibitory control. There were no effects of gamification on mood and user experience, despite a negative effect on perceived efficiency.

**Conclusions:**

The detrimental effects of heightened BMI on inhibitory control were preserved in a gamified version of the SST. Overall, the effects of overweight were smaller than in previously published web-based and laboratory studies. Gamification elements can impact behavioral performance, but gamified tasks can still assess inhibitory control deficits. Although our results are promising, according validations may differ for other types of behavior, gamification, and health variables.

## Introduction

Overweight and obesity are monumental health problems with dramatic increases in prevalence over the past centuries. A heightened BMI encompasses substantial risks for the onset of additional high-risk health conditions such as hypertension, diabetes, and cardiovascular diseases [1]. Next to complex genetic, biological, and environmental factors, and their interactions [2], a core behavioral phenomenon contributing to dysregulated energy balance in overweight is the degree of ability to control impulsive behaviors. Given the attractive value of particularly high-calorie food and its omnipresence in Western obesogenic environments, deficits in inhibitory control have been attributed as prominent factors for etiology and maintenance of energy imbalance behavior [3,4].

The stop-signal task (SST) is a hallmark adaptive paradigm for assessing inhibitory control that has been used extensively in basic research with healthy, overweight, and patient populations. The SST is a cognitive task that requires participants to cancel behavioral responses in a minority of trials after the presentation of a stop-signal, which is delayed according to a performance-contingent time interval. Inhibitory control deficits in this task have been identified in overweight and obese populations [3,4], overweight children [5], and patients with binge-eating disorder [6]. For instance, in the web-based study by Houben et al [4], participants with a BMI of 25 kg/m² or more had longer stop-signal reaction times (SSRTs) in an SST with food stimuli compared with normal-weight participants, which indicates weaker inhibitory control. Meta-analytic results suggested an underlying inhibitory control deficit in overweight and obese persons regardless of bingeing [3]. Accordingly, the SST has also been explored as a training to improve inhibitory control capacities and motivation to engage inhibitory control in critical situations, with the overall aim to provide a rehabilitative or even preventive instrument [7,8]. Initial results from laboratory studies were promising and demonstrated the possibility of weight loss following inhibitory control training [9-13], but long-term results of prolonged interventions are still outstanding [7,14].

Such cognitive tasks or trainings—behavioral tests that measure an underlying cognitive function such as inhibitory control, working memory, or cognitive control—are usually considered to be effortful, repetitive, or even frustrating [15]. Accordingly, these negative affective states might lead participants to disengage or terminate the training activity altogether [16]. Variation of the design elements employed in an SST could improve motivation and training outcomes particularly in upcoming long-term studies comprising multiple sessions. In fact, results of previous studies employing game elements in cognitive trainings indicated positive effects on performance and motivation in clinical [17] and healthy populations [15,18]. Consequently, the so-called gamification [19] of cognitive trainings has increased substantially in recent years [20]. From early on, gamification has been defined as “the use of game design elements in non-game contexts” [19]. Accordingly, gamification elements are a heterogeneous group of design elements covering the graphical presentation of a task, its fictional embedding, performance-contingent feedback, social elements, and other dimensions. By adding game elements to a nongame context, a more enjoyable, exciting, and compelling user experience is contemplated. Some of the most frequent gamification elements are a narrative, occasionally along with a social setting (eg, companion character), a graphical theme, and a scoring system with visual and emotional feedback [21,22].

Two previous studies tested assessments with gamified versions of the SST. In a web-based experiment, Lumsden et al [23] investigated whether scores or a theme environment would affect performance in the SST. However, no effects of gamified variants on attrition rates in an online sample were observed and SSRTs were overall comparable for all variants [23]. Moreover, participants rated the task version with scores as more enjoyable than a plain version and a theme version [23]. A second, more recent study implemented the SST in an endless runner scenario and found comparable performance in both task variants [24]. From these results, it was concluded that web-based gamified versions of the SST can be a potential advancement to applications in mental health. However, neither study investigated the assessment of inhibitory control deficits with a gamified SST in an overweight population.

In this study, we investigated whether the effects of overweight on inhibitory control would be preserved in a gamified SST. In order to gather the most sensitive results, we created 2 maximally contrasting conditions of the same underlying SST. When changing aesthetic and incidental task elements without varying the core mechanics, behavioral performance can still be affected due to higher distraction and motivation, which might affect assessments in different populations. For instance, inclusion of a scoring system in a working memory training negatively impacted performance [25], and use of visually more complex stimuli might affect the psychometric properties of behavioral assessments in game-like environments [26]. If a gamified version of an SST is intended to address inhibitory control deficits in overweight populations, an important requirement would be that the effect of overweight is still present in the altered assessment setting. Technically, comparable effects of BMI on SSRT would indicate external validity of the gamified task. To address this question, we developed 2 versions of an SST comprising food and control stimuli, and we tested whether the effects of overweight on SSRT were present in randomized groups of online testers with varying BMI who were randomly assigned to the gamified or nongamified version of our task. According to the results of Houben et al [4] and the meta-analysis by Lavagnino et al [3], we predicted higher SSRTs for overweight participants in a neutral environment but also in a gamified environment with the identical adaptive SST in both versions.

## Methods

### Participants

A total of 111 participants were recruited for the study. A priori, we determined the sample size in a power analysis. For reproducing a positive medium-sized association between inhibitory control and overweight (r ≈ 0.35; based on the online study of Houben et al [4]), at least 49 participants were required in each group (α = .05; 1–β = 0.8; 1-sided). An additional dropout rate of 10% was considered. Participants were randomly assigned to the groups (gamified, nongamified), and demographic characteristics are reported in the results section (age, gender, handedness, BMI, eating pathology). According to the high prevalence of overweight and obesity in the general adult population, we expected about 39% of our participants to be overweight. Participants were required to complete the study on a desktop computer, to be registered on the Prolific platform, and to be older than 18 years; there were no additional screening criteria selected.

The experiment was approved by the local ethics committee of the Leibniz Institut für Wissensmedien, Tübingen (LEK 2020-023). All complete submissions passed the fair attention check items implemented in the beginning and end of the study (see questionnaires). Recruitment used the Prolific platform, and all complete and valid submissions were reimbursed for study participation (3.60 £ [ca. US $5]; 72% of all submissions were valid; 5% of invalid submissions were rejected following the Prolific guidelines; 23% were returned or timed out by participants). The study took approximately 30 minutes.

### Gamified and Nongamified SST

**Figure 1:**
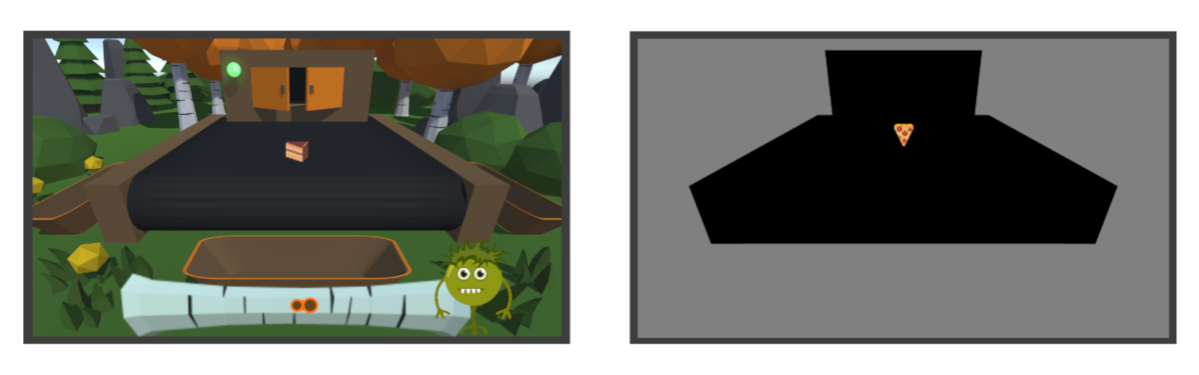
Left: The gamified environment. Stimuli appeared from the warehouse gate at the top of the conveyor belt and subsequently moved down the conveyor belt. The score bar and the companion character, Fred, are visible at the bottom of the conveyor belt. In case of correct responses, the score marker moved to the right; in case of wrong responses, the score marker moved to the left. Right: The nongamified environment. Only the stimuli remain the same as in the gamified version. A black shape covers the area covered by the conveyor belt and the warehouse gate. The forest environment is replaced by a grey background.

**Figure 2:**
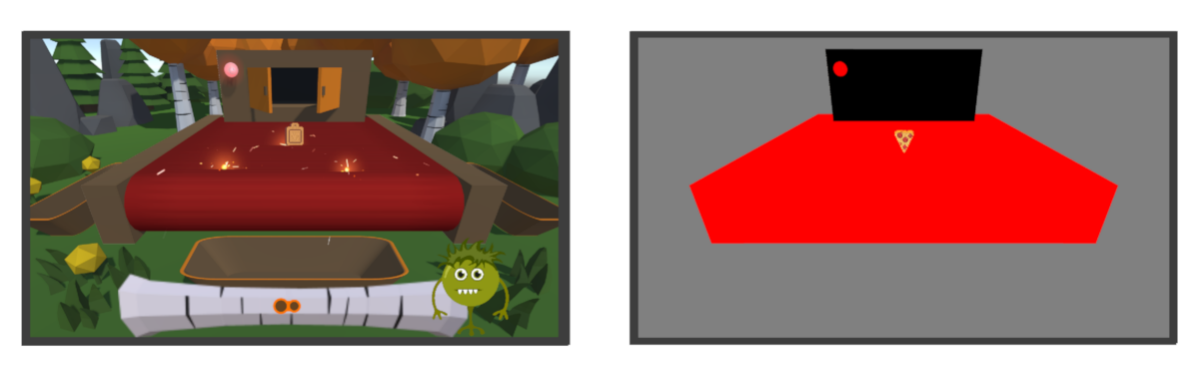
Left: Stop trials in the gamified version. After the stop-signal delay, the light at the warehouse gate and the conveyor belt turned red. Furthermore, the conveyor belt emitted sparks, resembling a short circuit. Right: Stop trials in the nongamified version. The area covered in red matches the location but is slightly larger than in the gamified version. Participants were requested to wait without responding until the end of the trial.

A gamified and nongamified version of the adaptive SST were newly developed for the purpose of this study (Figures 1 and 2). For reliable online assessment of behavioral responses, the task was implemented using the JavaScript library processing.js [27], along with additional code elements from jsPsych 6.0.4 for questionnaires. On the server side, the experiment was controlled with the JATOS framework [28], a Java environment that allows recording data, starting experimental modules, and creating links for participants to run the experiment. The experiment was run and data were stored on a server at the author’s institution in Tübingen, Germany.

The gamified and nongamified SSTs were identical with regard to the task procedure. In order to maximize possible effects, the gamified SST additionally included a narrative and graphical theme, scoring system with visual and emotional feedback, and a companion character. These additional gamification elements are described in detail in the next section.

We adhered to the recent consensus recommendations for designing and reporting SST performance [29]. Participants performed a 2-alternative forced choice task, where they had to discriminate objects based on their color. Participants were instructed to press a left or right key (A or L) as fast as possible in go trials but cancel their response in stop trials. We used a bold and salient stop-signal, realized in terms of a red color cue that covered over 25% of the 960×540 screen. In order to avoid waiting strategies and in line with the recommendations, only 25% of the trials were stop trials. Following Verbruggen et al [29], we used an adaptive tracking procedure to adapt stop-signal delays (SSDs). The initial SSD for all participants was set to 200 ms. In cases where participants responded incorrectly in stop trials—including premature responses—the SSD was decreased by 50 ms, down to a minimum of 50 ms. In cases of successful stop trials, the SSD was increased by 50 ms up to a maximum of 900 ms. We also instructed participants explicitly not to wait. The exact wording was “You must not wait for the color change [the stop-signal], otherwise your response times are very long.” Participants could familiarize themselves with the procedure in training trials; however, we did not provide blockwise feedback. This was due to the fact that we conducted an online study and tried to keep the experiment as short as possible, with only one single block (and self-paced breaks). In the main experiment, participants performed 256 trials, and 64 of them were stop trials. According to the computational models from Verbruggen et al [29], 50 stop trials should suffice to obtain unbiased stop-signal reaction times (SSRTs).

### Gamification Elements

The gamified SST was developed in a low-poly graphical style. This design was chosen for two reasons. First, it is considered aesthetical particularly in game-like environments and resembles the style of previous gamified versions of the SST [11,24]. Second, due to its popularity in game design, a lot of free assets are available, like the low-poly nature assets used here.

The displayed environment resembled a forest glade surrounded by trees and rocks. A conveyor belt was in the center of the glade. On top of the conveyor belt, a warehouse gate with a light bulb on the left side was shown (see Figure 1, left panel). At the beginning of each trial, the gate opened, the light bulb turned green, and a stimulus spawned at the top of the conveyor belt. Stimuli traveled down the conveyor belt (this animation was shown in both versions) until participants pressed the left or right key. In this case, the stimulus flew to 1 of 2 bins beside the conveyor belt (only in the gamified version). If participants did not respond, the stimulus fell into a bin at the end of the conveyor belt. When landing in a bin, the stimulus disappeared and a sparkling effect was presented. A trial ended when the stimulus landed in either one of the bins.

During go trials, the light bulb stayed green during the whole trial. In stop trials, the light bulb and conveyor belt turned red (this animation was shown in both versions) Furthermore, the conveyor belt emitted sparks, resembling a short circuit (see Figure 2, left panel, shown only in the gamified version). In this case, participants should not respond at all but should wait until the stimulus fell off the conveyor belt.

Below the conveyor belt, a score bar was displayed on top of a wood log. In case of correct responses, the score indicator moved to the right, in case of wrong answers or responses during stop trials, the bar moved to the left.

To the right of the bar a cartoon figure (Fred) was shown, whose facial expression depended on the score (ie, the figure showed a neutral expression for scores between 25% and 75%, a sad expression for scores below 25%, and a happy expression for scores above 75%). The cartoon figure was also used to provide a narrative theme for the experiment. Participants were requested to help the color-blind Fred operate the conveyor belt by sorting red objects to one bin and brown objects to the other. Stop trials were introduced by mentioning that the conveyor malfunctions sometimes, and participants should not respond in these cases. Participants were also encouraged not to wait for a malfunction, as Fred would not be productive enough in this case.

To sum up the gamification elements, we used a scoring system with visual and emotional feedback, a brief narrative, and appropriate graphics. For the nongamified SST, all elements were removed and only the stimuli remained the same. Instead of the forest glade, participants saw a monochrome grey background. The warehouse gate and conveyor belt were replaced with black shapes covering the very same area. The light bulb remained as a colored circle, which stayed green in go trials and turned red in stop trials. The black shape covering the area corresponding to the conveyor belt in the gamified SST turned red in stop trials. There were no bins, no animations, and no score bar in the nongamified SST (see Figure 1, right panel and Figure 2, right panel).

### Stimuli

**Figure 3:**
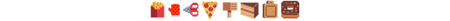
The stimuli. Left: 4 red targets—French fries, glove, scissors, and pizza. Right: 4 brown targets—wooden hammer, cake, chopping board, and donut. Pixel art stimuli were selected for their low-poly, 8-bit style. Stimuli were created by artist VectorPixelStar [30] and used in agreement with the artist in this study.

Participants solved a color decision task in go trials, pressing 1 of 2 defined buttons (A or L) for red or brown targets. The stimuli were selected to match the low-poly environment and consisted of pictograms of high-calorie food (donut, pizza, cake, French fries) or approximately color- and tone-matched nonfood kitchen objects (glove, scissors, wooden hammer, chopping board; see Figure 3). Accordingly, in order to create an overall consistent ambience in the game condition, no realistic pictures were used as these might break with the virtual character and remainder of the visuals shown. Target presentation within the SST was randomized. The factor stimulus type was included in our analysis, because previous studies observed effects of overweight only in blocks of food stimuli [4].

### Questionnaires

#### Positive and Negative Affect Schedule

To assess participants’ affective states, the Positive and Negative Affect Schedule (PANAS) was employed [31,32]. Both positive (PA) and negative affect (NA) are measured by 10 items. Participants must indicate how they are feeling at the moment by rating items on a Likert scale from 1 (very slightly or not at all) to 5 (extremely).

#### User Experience Questionnaire

The User Experience Questionnaire (UEQ) [33] was used to assess participant user experience. The questionnaire uses bipolar ratings from 1 to 7 (eg, not interesting to interesting) and evaluates general attractivity, as well as *hedonic quality* (stimulation, novelty) and *pragmatic quality* (efficiency, perspicuity, dependability) of the software.

#### Game Preferences Questionnaire

The Game Preferences Questionnaire (GPQ) [34] includes 1 item to assess gaming frequency on a Likert scale from 1 (never) to 7 (daily). Further, 9 items are used to measure preferences for certain video game genres (eg, first person shooter, strategy games, adventure games) on a 7-point Likert scale from 1 (strongly dislike) to 7 (strongly like).

#### Dutch Eating Behavior Questionnaire

The Dutch Eating Behavior Questionnaire (DEBQ) was used to evaluate general eating pathology [35]. The DEBQ consists of 33 items regarding eating behavior and eating-related cognitions, which are divided into restrained eating, emotional eating, and external eating items.

#### Hunger Visual Analog Scale

A single item asked participants for their current hunger state before the task was started (ie, “How hungry are you in this moment?”). Participants moved a slider between the anchors not hungry and very hungry in response.

#### Attention Checks

Several fair attention checks items [36] were distributed across the scales before and after the SST. In total, dependent on the question length, there were 6 items such as “To show that you pay attention, please write 2020 in the field below” (eg, as open text field), “To show that you pay attention, please select ‘very often’ here” (eg, as an item within the DEBQ with the options “never,” “seldom,” “sometimes,” “often,” and “very often”) or “click ‘extremely’ please” (eg, as an item within the PANAS with a Likert scale from 1 [very slightly or not at all] to 5 [extremely]). The aim of these items was to confirm that participants actively read instructions and questions, thus ensuring data quality. All fair attention checks were passed by the included study participants in both groups. None of the full study submissions needed to be excluded based on these items.

#### Additional Questionnaires

For exploratory purposes, we also collected responses on the Body Shape Questionnaire [37] and an adapted version of the Implicit Theories of Intelligence Scale [38]. However, as these constructs were not relevant for answering the research questions of this study, analyses using these questionnaires are not reported here.

### General Procedure

Participants were randomly assigned to the gamified or nongamified SST. Beside the task, both groups completed several questionnaires. Before starting the study, participants completed a captcha by drawing a certain path. Next they received general information about the procedure of the study; after this, they were asked to provide their year of birth and complete a PANAS questionnaire. This was followed by the first attention check and instructions for the SST. Participants then completed the gamified or nongamified SST. After this, participants were requested to complete several questionnaires, starting with a second PANAS and a UEQ. This was followed by demographic data (age, gender, handedness, BMI, eating pathology), a second attention check, GPQ, DEBQ, body shape questionnaire, and implicit intelligence questionnaire.

### Data Treatment and Statistical Analysis

Out of the approved submissions with correctly answered attention checks, data inspection revealed 1 additional submission without behavioral data (failed recording of reaction times) and 3 submissions with self-reported height below the 95% confidence interval of human height [39], which could not be considered for calculation of BMI effects. These submissions were reimbursed but not further considered for analyses. Next, the prerequisites for the estimation of SSRT were evaluated [29]. Responses of 7 participants were excluded because stopping probabilities were outside of the interquartile interval (25% to 75%) in 1 or 2 conditions. Moreover, latencies in stop trials were longer than latencies in go trials for 11 participants. In line with the recommendation of Verbruggen et al [29], these values were rejected from the analyses as the assumption of an independent race model was violated.

For investigation of mean reaction times, only responses from go trials were considered. Mean reaction times were aggregated individually and separately for food and neutral stimuli. For investigation of inhibitory control, the SSRT was calculated following the integration method with replacement of go omissions [29]. Since the time for canceling a response cannot be measured directly, this approach considers the individual stopping probability to determine mean reaction time. Next the mean SSD was calculated for every condition and subtracted from reaction time (SSRT = RT[go|pStop] – mean[SSD]). This procedure yields the most reliable SSRT estimate [29]. In the task, SSD was adjusted separately for food and neutral stimuli; accordingly, both SSRT measures could be calculated.

To account for the imbalanced data set resulting from the data cleaning, we used linear mixed effects modeling for the analyses [40]. All analyses were performed in R version 4.0.3 (R Foundation for Statistical Computing) [41], using the nlme package [42]. Subjects were modeled as random effects, with *stimulus* (food vs control) as repeated measures fixed effects and *overweight* (low vs high) and *group* (gamified vs nongamified) as between subjects fixed effects. In line with Houben et al [4], overweight status was operationalized as BMI **≥**25 kg/m², which led to comparable proportions of approximately 30% overweight and obese participants in both groups (see Table 1). Moreover, age was entered as a continuous variable to the models following the significant age difference between the gamified and nongamified group. To quantify effect sizes with continuous variables, product-moment correlation coefficients were calculated.

## Results

### User Statistics

The groups were comparable in a number of demographic variables (Table 1, Multimedia Appendix 1). Importantly, there were neither significant differences in BMI nor general eating pathology according to the DEBQ. However, a significant age difference was observed (t_78.47_=2.29, *P*=.02) with slightly older age in the game group (29.6 [SD 11.3] years) than in the no-game group (25.3 [SD 7.2] years). To control for age differences in behavioral scores, particularly regarding inhibitory control, age was entered as covariate to the subsequent models.

Gaming frequency was moderate in both groups (see Table 1). Following the suggested use recommendation of the GPQ [34], participants were classified into 4 groups of gamers (casual = 16, well-rounded = 9, hardcore = 28, no-gamer = 47). That is, about half of the participants can be considered to be no-gamers.

**Table 1:** User statistics for the gamified and nongamified group.

Characteristic	Gamified (n=48)	Nongamified (n=52)
Overweight, n (%)	15 (31)	15 (29)
BMI, normal weight, mean (SD)	21.42 (1.92)	21.14 (2.61)
BMI, overweight, mean (SD)	29.28 (3.11)	27.97 (4.05)
State hunger, mean (SD)	37.85 (31.62)	38.46 (31.63)
DEBQ^a^ restrained, mean (SD)	16.96 (8.83)	17.08 (9.74)
DEBQ emotional, mean (SD)	20.90 (12.39)	20.38 (12.93)
DEBQ external, mean (SD)	22.63 (5.71)	23.54 (6.24)
Female [other], n (%)	22 (46) [0 (0)]	18 (37) [2 (4)]
Left-handed, n (%)	5 (10)	8 (15)
Age in years, mean (SD)	29.6 (11.3)	25.3 (7.2)
Gaming frequency, mean (SD)	3.23 (2.12)	3.17 (1.98)

^a^DEBQ: Dutch Eating Behavior Questionnaire.

### Reaction Times

For mean reaction times in go trials, the covariate *age* was not significant (*F*_1,95_=0.12, *P*=.74). The main effects of *condition* (*F*_1,95_=48.87, *P*<.001) and *overweight* (*F*_1,85_=6.63, *P*=.01) were statistically significant. Moreover, there were significant 2-way interactions between *overweight* and *condition* (*F*_1,85_=3.98, *P*=.049), as well as between *overweight* and *stimulus* (*F*_1,87_=4.40, *P*=.04).

**Figure 4:**
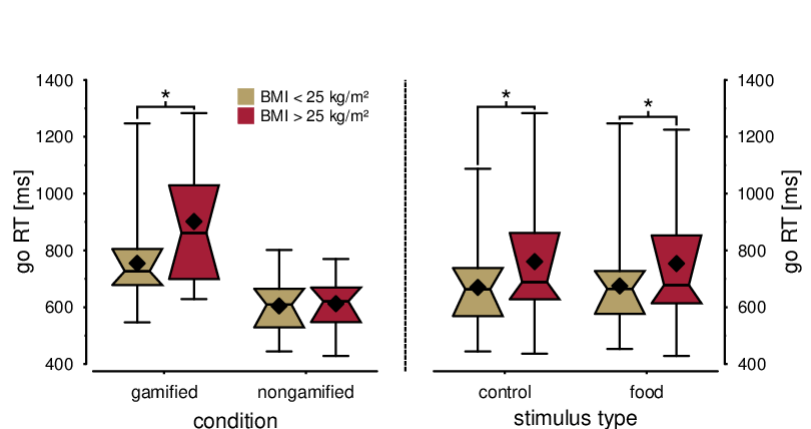
Effects of gamified versus nongamified stop-signal task and control versus food stimulus type on mean reaction times in go-trials. Longer reaction times of the overweight group were observed in the gamified stop-signal task but not in the nongamified stop-signal task. This effect was slightly larger for control stimuli; there was no significant stimulus difference within or across groups. RT: reaction time.

Figure 4 illustrates these interactions. Longer mean reactions were particularly pronounced in overweight participants in the game condition (*F*_1,45_=5.79, *P*=.02), but not in the nongamified condition (*F*_1,45_=0.26, *P*=.61; Figure 1, left panel). Moreover, prolonged responses of overweight participants were particularly pronounced for control stimuli (*F*_1,90_=9.73, *P*=.002), but the difference between groups was still significant for food stimuli (*F*_1,91_=6.92, *P*=.01). Further interaction terms or the stimulus main effect were not statistically significant (*F*s<1.23, *P*s>.27).

### SSRTs

For SSRTs, the covariate *age* was statistically significant (*F*_1,95_=4.93, *P*=.03), and SSRTs increased with older age (*r*_189_=0.25, *P*<.001). Moreover, there was a significant main effect of *condition* (*F*_1,95_=33.83, *P*<.001), indicating longer SSRTs in the gamified condition (383 [SD 115] ms) compared with the nongamified condition (294 [SD 48] ms). In line with our prediction, a trend was observed for the main effect *overweight* (*F*_1,95_=3.39, *P*=.07), driven by slightly longer SSRTs in overweight participants (361 [SD 123] ms) compared with normal weight participants (328 [SD 84] ms).

**Figure 5:**
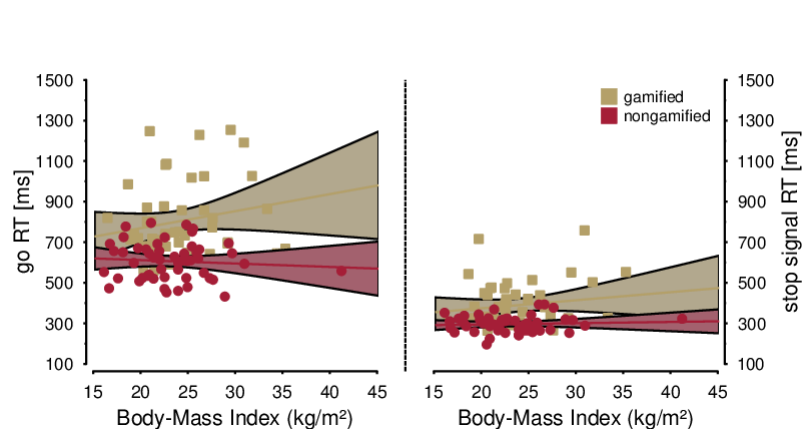
Scatter plots of BMI as a predictor for go reaction time (left) and stop-signal reaction time (right). The gamified versus nongamified condition qualified the association with mean go reaction time but not with stop-signal reaction time. RT: reaction time.

Most importantly, *overweight* was not further qualified by an interaction with the factor *condition* (*F*_1,95_=0.16, *P*=.69) nor by any other 2- or 3-way interaction (*F*s<1.17, *P*s>.28). Across the entire sample, SSRTs increased with BMI (*r*_189_=.155, *P*=.03). Notably, the effect size was substantially smaller than in previous research (see Figure 5, right panel).

As a sensitivity analysis, we also calculated the correlation coefficient after exclusion of outlier values, as indicated by visual inspection of the scatter plot (Figure 5). Six values were identified as outliers. The magnitude and significance of the overall associations did not change substantially (*r*_183_=.162, *P*=.03).

### Effects on Mood and User Experience

Both positive and negative affect decreased from pre-SST to post-SST in both conditions (*F*_1,98_=23.74, *P*<.001), but there was neither an interaction with valence (*F*s<1.97, *P*s>.17) nor any interaction with the gamified or nongamified condition (*F*s<1.40, *P*s>.24). In the post assessment of the task with the user experience questionnaire, no significant differences were observed for attractiveness, perspicuity, dependability, stimulation, or novelty (*t*s<1.52, *P*s>.15). The efficiency score of the gamified SST (11.41 [SD 2.62]) was significantly lower than the nongamified SST (12.60 [SD 1.97]; t_87.01_=–2.52, *P*=.01).

## Discussion

### Principal Findings

This study investigated the effects of game elements in an SST in participants with and without overweight. Our primary aim was to investigate whether inhibitory control deficits were preserved in a gamified SST. We observed worse performance and inhibitory control in overweight participants, and effects of overweight were preserved or even enhanced in the game variant of the SST. Overall, these results indicated that a gamified SST can be used to assess inhibitory control deficits in overweight populations, and we replicated effects of overweight on inhibitory control. This suggests the external validity of using a gamified version of the SST.

Results of this study showed that the gamified SST was more difficult than a more conventional version of the SST without game elements as reflected by higher go reaction times, which was particularly apparent in overweight participants. The additional game elements in the gamified version of the SST might have distracted participants and increased participants’ cognitive load, which resulted in longer reaction times. This seems to be in line with research on so-called seductive details—interesting but irrelevant elements for achieving a task’s objectives. As such, research in the field of seductive details showed that, for instance, adding pictures to learning material such as textbooks that are not strictly relevant for achieving the instructional goal but rather should make the learning material more interesting can lead to poorer performance (for a review see Rey [43]). In this case, for instance, the additional presence of the character, Fred, with animated feedback might have drawn the attention of participants and slowed their overall performance. Indeed, results of a recent eye-tracking study comparing a gamified with a nongamified task indicate that the game character provided was one of the most frequently fixated game elements [44]. The pattern of results could imply that the gamified task might recruit additional cognitive mixed abilities [26]. Accordingly, it is crucial to investigate the external validity of the measures from the task with other external variables such as weight status (eg, if the task is more complex and distracting, it could be that its outcome measures such as SSRT or mean reaction time would not only reflect a specific cognitive function but are affected by additional involvement). Accordingly, a gamified task could in theory no longer measure deficits in a particular way.

Because multiple game elements like an additional narrative, graphical theme, scoring system with visual and emotional feedback, and a companion character were incorporated in the gamified version of our study, it is not clear which of these elements exactly led to overall longer reaction times. Previous research could potentially indicate that the theme manipulation, which usually includes extensive changes to graphics, might be particularly distracting; in a study comparing points and themes individually, the themes variant of an SST was evaluated as worst but had also descriptively less attrition, paradoxically [23]. That study also showed comparable behavioral results (ie, longer reaction times in a theme vs neutral variant) with slightly smaller effect sizes, suggesting that the additional game elements might have exacerbated the effects on performance in this case. In a separate study, performance was improved by points, but enjoyment suffered in a themed variant of another task [45]. From our results, we can only speculate that the possible motivating effects of a score bar might have been abolished by the presentation of a graphical and narrative theme, but future research is required to investigate the isolated and combined effects of these design elements. Since the goal of our study design was to create maximally contrasting conditions, the general behavioral effects in reaction times confirm that the sum of gamification elements indeed affected performance.

Concerning participants’ inhibitory control, SSRTs were slower in the gamified compared with the nongamified version of the SST. This again might indicate an increased perceived cognitive load due to the implemented game elements. Alternatively, it could also be related to the dependence of SSRT on go reaction time distributions. Most importantly, the gamified task condition did not interact with overweight status of the participants, and we observed a slight yet nonsignificant increase in SSRTs with heightened BMI, in the direction of our prediction. This effect was substantiated by a positive association between SSRTs and BMI across task conditions, even though the effect was smaller as in previously published studies [3,4]. Nevertheless, this study replicated inhibitory control deficits in overweight populations, suggesting an underlying cognitive deficit [3,4]. Moreover, the results also demonstrated the heightened difficulty with exerting inhibitory control in visually more complex situations (ie, the use of game elements). Importantly, since associations of SSRTs with BMI in the gamified SST were preserved, we suggest it appears promising to use such settings for future inhibitory control trainings. Besides potential effects of game elements on performance and motivation, a (visually) more rich, naturalistic, or diversified training environment might facilitate transfer effects to inhibitory control behavior, although the available evidence on this hypothesis is still very scarce [7,46]. Food-specific response inhibition deficits are already present in overweight elementary school children [5], and gamified response inhibition trainings could be particularly promising in younger populations.

Additional interesting observations were obtained in the analysis of go trial reaction times. Beyond the main effect of condition, overweight participants’ responses were particularly slower in the gamified SST, whereas there were no significant group differences in go reaction times in the conventional SST. Moreover, the pattern of increased go reaction times in overweight participants was slightly larger for neutral stimuli but still significant for food stimuli. This might reflect higher response (motivational) salience of food cues in overweight participants [47]. However, other studies in a go-/no-go task observed similar trends for faster responses to food cues in both obese and healthy participants [48] or opposite effects of faster responses to neutral cues in a conventional SST [49]. To investigate these inconsistencies in the literature, the exact configurations of tasks, stimuli, and other study design characteristics should be addressed in future studies. Nevertheless, a more straining task using a visually richer and distracting environment, at least compared with the conventional versions of the SST, might be more comparable to inhibitory control requirements in complex and distracting real-life settings [7].

By implementing game elements into the SST, we aimed at increasing mood and user experience of participants. Our analyses, however, showed that in both the gamified and nongamified SST, positive and negative effect decreased from pretest to posttest. Despite the use of supposedly (emotionally) engaging game elements, changes in mood were comparable in both conditions. This is in contrast to previous studies showing that game elements increase positive affect [50] or at least help to prevent positive affect from dropping in strenuous cognitive tasks [15]. Moreover, attractiveness of the task design and hedonistic and (most) pragmatic qualities of user experience were not affected by the used game elements. On the contrary, the gamified SST was rated to be less efficient than the nongamified SST. This might suggest a less than optimal integration of game elements in this experiment. Consequently, the use of game elements in the current implementation did not prevent negative affective effects of performing a cognitive task usually considered to be cognitively challenging. Interestingly, a previous similar theme- and point-based implementation of the SST had equal attrition rates compared with a neutral variant [23]. In sum, these results may suggest that a gamification of this task in an online setting might require further design changes.

### Comparison With Prior Work

Previous studies had investigated assessments with a gamified SST [23,24]. Lumsden and colleagues [20] investigated whether scores or a theme would affect attrition rates and performance in a web-based SST across multiple sessions of testing. Compared with a neutral version of the SST, no differences were observed regarding attrition, which suggests similar motivation of participants. In their study, points affected users’ engagement positively in the subjective evaluations. Regarding inhibitory control, SSRTs in the theme variants of their SST were higher than SSRTs in the point variant (but statistically not different from the neutral variant; see Figure 12 in Lumsden et al [23]), which appears to be consistent with our results. Moreover, their study also showed higher mean reaction times in the theme condition compared with the neutral variant (supplementary material in Lumsden et al [23]).

More recently, Friehs and colleagues [24] designed and developed a more sophisticated version of the SST by using an endless runner scenario in a 3D virtual environment. While participants performed similarly in both the gamified and nongamified versions, the gamified version led to higher enjoyment and flow. This might indicate that more substantial changes to the SST, as realized by Friehs and colleagues [24], are necessary to change participants’ affective states. Contrary to our results, there were no performance differences between the task versions [24].

Moreover, whereas some previous studies observed cue-specific effects of food stimuli [3,4,48], the factor stimulus-type was not significant in other experiments [49] or in our study. We consider various potential factors of interest for further investigation: (1) our low-poly stimuli may have been too abstract (as opposed to photographs of real food), (2) both stimulus types appeared in the same SST blocks, with separate calculation of SSRT (but see Svaldi et al [6]), (3) the stimulus category was not task relevant, and (4) the control category was topically related to food cues. For training studies, cue-specific stimuli appear to be highly relevant [7].

### Limitations

This internet study assessed BMI in self-report, analogous to previous studies [4]. Nevertheless, self-presentation biases might have been evident in both groups. The subjective evaluations of the gamified SST in the UEQ were weak, since mood decreased in both conditions and no differences in user experience were appraised by our study participants. Thus, there is potential for improvements in the user experience of the gamified SST. As outlined by a reviewer, more nuanced and implicit assessments of user enjoyment should be considered in future research (eg, dual tasks or attrition rates [23,51]). However, we found relatively large performance differences in the 2 versions, comparable to the experienced differences in efficiency, with higher values for the no-game task. Since we recruited clickworker for a single assessment study, the potential role of self-motivation in an SST training could not be considered (but see Forman et al [13] and Lumsden et al [23]). Change motivation (eg, concurrent participation in a weight-loss program) and psychoeducation could substantially increase the user experience in future studies.

Participants in this study had no formal diagnosis and we did not control for patterns of binge eating, in line with previous research [4]. General eating pathology in the gamified and nongamified groups was comparable according to the DEBQ [35]. Finally, participants in the gamified SST were significantly older than participants in the conventional SST group.

### Conclusions

We observed longer reaction times in the gamified task, particularly in overweight participants, and longer SSRTs. The detrimental effects of heightened BMI on inhibitory control were preserved in a gamified and comparable nongamified version of the SST, as shown in small positive associations of overweight with SSRTs regardless of task version and stimulus type. However, mood and user experience were identical in both version; thus, it seems that the design of an enjoyable version of this task remains difficult. Gamification elements can impact behavioral performance but can be used to assess inhibitory control deficits in different populations.

## Appendix

Multimedia Appendix 1 Comparison of overweight and normal weight groups.

## Abbreviations

DEBQ: Dutch Eating Behavior Questionnaire
GPQ: Game Preferences Questionnaire
PANAS: Positive and Negative Affect Schedule
SSD: stop-signal delay
SSRT: stop-signal reaction time
SST: stop-signal task
UEQ: User Experience Questionnaire
