# Supplementary material for: Preserved Inhibitory Control Deficits of Overweight Participants in a Gamified Stop-Signal Task: Experimental Study of Validity
Source: JMIR Serious Games. 2021 Mar 12;9(1):e25063. doi: 10.2196/25063 (PMC8092187; doi:10.2196/25063)
Supplement: Multimedia Appendix 1 [file games_v9i1e25063_app1.doc]

**Multimedia Appendix 1.** Comparison of Overweight and Normal weight groups. Differences between BMI and Restrained Eating subscale of the DEB-Q were statistically significant.

|  | Overweight | Normal weight |
| --- | --- | --- |
| N | 30 | 70 |
| BMI | 28.62 (3.61) | 21.27 (2.30) |
| State Hunger | 32.90 (26.49) | 40.42 (33.29) |
| DEBQ Restrained | 21.93 (7.56) | 14.91 (9.18) |
| DEBQ Emotional | 23.37 (13.81) | 19.46 (11.98) |
| DEBQ External | 22.90 (5.37) | 23.19 (6.25) |
| % female (other) | 30 (3) | 44 (1.4) |
| % left-handed | 13.3 | 12.9 |
| Age | 28.1 (10.4) | 27.0 (9.14) |
| Gaming Frequency | 3.47 (1.85) | 3.09 (2.11) |
